# The Treatment of Opsoclonus-Myoclonus Syndrome Secondary to Neuroblastic Tumours—Single-Centre Experience and Literature Review

**DOI:** 10.3390/medicina56080412

**Published:** 2020-08-14

**Authors:** Agnieszka Mizia-Malarz, Weronika Stolpa, Grażyna Sobol-Milejska

**Affiliations:** Department of Pediatric Oncology, Haematology and Chemotherapy, Upper Silesia Children’s Care Heatlh Centre, Medical University of Silesia, 16 Medykow Street, 40-752 Katowice, Poland; wera1pl@poczta.onet.pl (W.S.); grazynasobol@o2.pl (G.S.-M.)

**Keywords:** opsoclonus-myoclonus syndrome, neuroblastic tumours, treatment, children

## Abstract

*Background and Objectives*: The opsoclonus-myoclonus syndrome (OMS) is characterised by opsoclons, myoclons and impaired balance, often concomitant with sleep disorder and behavioural difficulties. The symptoms develop as a result of autoimmune response triggered by a neuroblastic tumour (NT). OMS can also develop secondarily to a viral infection or as an immune response triggered by an unknown agent. This leads to the activation of B- and T-cells, which produce and release autoantibodies or cytokines directly within the central nervous system (CNS), thus damaging the neurons within the cerebellum and the brain stem. The available OMS treatments aim at decreasing lymphocyte, cytokine and autoantibody production or accelerating the utilisation of the latter. Another treatment option for OMS involves using cytostatic agents, which damage T- and B-cells causing their depletion and impaired function, which reduces their ability to produce antibodies and cytokines. *Materials and Methods*: We present a single-centre experience in treating OMS secondary to NT in 7 children. *Results*: The combined treatment with cyclophosphamide plus dexamethasone resulted in a complete resolution of OMS symptoms in 4 children, and a significant improvement in the 3 children. Two of them periodically present hyperactivity, and one girl requires an additional support at school due to special educational needs (SEN). *Conclusions*: NT resection does not resolve OMS in children with OMS secondary to NT. The combined treatment with dexamethasone plus cyclophosphamide seems to be an effective treatment of OMS.

## 1. Introduction

Opsoclonus-myoclonus syndrome (OMS) is a clinical syndrome of an undetermined, most likely autoimmune, aetiology [1,2,3,4,5]. It is characterised by opsoclony, myoclony and impaired balance, often concomitant with sleep disorder and behavioural difficulties. It affects young children at the mean reported age of 1.5–2 years [6,7,8]. OMS is considered a paraneoplastic syndrome. Approximately 2–3% of children with a known malignancy present OMS. In 40–80% of paediatric patients with neuroblastic tumour, such as neuroblastoma, ganglioneuroblastoma or ganglioneuroma, opsoclonus-myoclonus syndrome could be the first sign of this type malignancy [6,7,9]. OMS may also develop as a result of a viral CNS infection or an autoimmune condition [6], without a concomitant tumour.

Its aetiology has been poorly understood. In cases with concomitant NT, the bodily response most likely utilises defence mechanisms against onconeuronal antigens present on the NT cells. This leads to the activation of B- and T-cells, which produce and release autoantibodies or cytokines directly within the central nervous system (CNS), thus damaging the neurons within the cerebellum and the brain stem [1,10,11,12,13].

The available OMS treatments, such as glucocorticosteroids (GS), intravenous immunoglobulins (IVIG) or anti-CD20 antibodies, aim at decreasing lymphocyte, cytokine and autoantibody production or accelerating the utilisation of the latter. Cytostatic agents, used in OMS treatment, damage T- and B-cells causing their depletion and impaired function, which reduces their ability to produce antibodies and cytokines [1,3,14,15,16]. There is anecdotal evidence of using plasmapheresis in OMS [17].

## 2. Material and Methods

Forty-seven children were diagnosed with neuroblastic tumors (NT) between January 2006 and December 2017 in our Department. Seven of them (14.9%) presented OMS. These were 2 boys and 5 girls with a mean age of 27 months (range 14–36 months). Prior to NT diagnosis, all 7 children diagnosed with OMS reported presented neurological symptoms in keeping with OMS (opsoclonus-myoclonus syndrome) of variable severity. The symptom duration until the tumour diagnosis ranged between 3 weeks and 8 months. The group characteristics are presented in Table S1.

All patients’s guardians had the need for treatment explained to them. Applied pharmacological therapy in children is used in patients with autoimmune processes.

The treatment started with a total resection in four of seven children. Due to persistent OMS, glucocorticosterioids were used (prednisone, at the dose of 2 mg/kg for 4 weeks with subsequent dose tapering), followed by intravenous immunoglobulins (0.4 g/kg for 5 days). One of these children underwent two courses of 4-week steroid therapy, as described above.

Three of seven children had a preliminary diagnosis of acute cerebellar ataxia. Two of them started prednisone (dosage regimen as described above), whereas one underwent the immune therapy. Due to non-response, magnetic resonance imaging (MRI) was performed, which demonstrated retroperitoneal tumours in the 3 children. A total resection was performed in 2 cases and a subtotal resection in 1 case.

All 7 children did not respond well to the above treatment. A mild reduction of OMS symptoms was achieved in four of seven cases, with no response to the isolated steroid and immune therapy in three of seven remaining cases.

Having discussed the available treatment options with the parents/carers, the decision to start a combined treatment with cyclophosphamide plus dexamethasone on 6 of the patients was made. This 12 month treatment regimen included cyclophosphamide 750 mg/m^2^ on day 1, plus dexamethasone pulses of 20 mg/m^2^ on days 1, 3 in months 1, 6, 8, 10 and 12, and dexamethasone pulses of 20 mg/m^2^ on days 1, 3 in months 7, 9 and 11. The cumulative dose of cyclophosphamide was 6750 mg/m^2^ in 5 cases. In one girl who presented severe symptoms upon admission and was slow to respond to the treatment, the above regimen was continued for an additional 4 months; the cumulative dose of cyclophosphamide was 8250 mg/m^2^. The steroid therapy was additionally prolonged in two children, mostly due to the parental fear of a possible recurrence. They were administered four low-dose dexamethasone pulses at one-month interval. A clinical improvement and good tolerance were observed in all the children.

## 3. Results

A complete remission (CR) of the malignancy was achieved in six of the seven children. In one female, after a subtotal resection of the paraspinal tumour diagnosed as neuroblastoma differentiating type, n-myc (−), chemotherapy was used (etoposide + carboplatin/cyclophosphamide + doxorubicin + vincristine), which resulted in a complete resolution of OMS and a complete remission unconfirmed (Cru) of the malignancy. A complete remission of OMS was achieved in 4 of seven 7 children. A total of 2 of 7 cases periodically manifest hyperactivity, especially a girl with an intellectual disability who shows the most severe and persistent symptoms. One child, after getting burnt and getting a viral infection, periodically manifests myoclonus. Currently, this child is in complete OMS remission. The follow-up of these patients is included in Table S1.

## 4. Discussion

OMS rarely occurs in patients with NT. Only approx. 2–3% of children with known NT present OMS [3]. The estimated incidence of concomitant NT and OMS is 0.03 cases per million in the USA, 0.18 cases per million in the UK and 0.27–0.4 cases per million in Japan [4,7,8]. In a study by Takama et al. [3], 5 of 73 children with NT were diagnosed with OMS over a 12-year period. In our 11-year time observation, this incidence rate is noticeably higher (14.9%).

As the underlying mechanisms of OMS have been poorly understood, it is difficult to choose an optimal treatment. The most likely explanation involves an immune activation which results in an elevated count of T- and B-cells followed by their enhanced production of cytokines and immunoglobulins [1,10]. Therefore, in line with the current understanding, glucocorticosteroids, adrenocorticotropic hormone (ACTH) and intravenous immunoglobulins are the most commonly used in OMS treatment following NT resection, which is an integral part of the treatment, albeit insufficient as a stand-alone procedure [1,2,3,4,5,6,7,14].

A number of authors reported the steroid therapy as a method of choice in new OMS cases after NT resection (2, 3). In a group of 23 children with OMS, Tayoshima et al. [2] demonstrated a resolution of neurological deficits after a long-term treatment with GS and ACTH. Takama et al. [3] reported the need to use steroids and intravenous immunoglobulins in 4 of 5 patients after initial NT resection. One patient was additionally administered rituximab. Similarly, Krug et al. [18] reported the need to use steroids in 6 of 22 patients after initial NT resection. According to Takama et al. [3] and Tayoshima et al. [2], the intravenous immunoglobulin treatment is less effective and did not show high efficacy in Japanese patients. In our patients, a total resection followed by an isolated steroid or immune therapy did not resolve the symptoms.

Immunophenotyping of CSF lymphocytes in OMS may help guide the treatment. However, it is not commonly used. An elevated B-cell count would support the anti-CD20 antibody treatment with rituximab or ofatumumab in patients with known rituximab allergy [4,19]. Both agents cause B-cell depletion in CNS, which decreases the antibody count and resolves OMS symptoms [2,12,14,15,17]. As pointed out by Tayoshima et al. [2], rituximab offers benefits not only in the first line therapy but also in recurrent OMS. However, using anti-CD20 antibodies in patients with OMS is not supported unequivocally. According to Chang et al. [20], immunosuppresion with rituximab may promote the progression of undiagnosed malignancy. According to other authors, low or undetectable B-cell count may indicate no need for the anti-CD20 antibody treatment. Starting rituximab on such patients may bring out more harm than benefit, considering possible adverse effects [1,2,4,14,19].

An elevated CD 8(+), HLA DR (+) T-cell count in the CSF with a reduced count of CD 4(+) T-cells normally abundant in CSF may indicate an involvement of T-cells in OMS [1,15]. Despite this understanding, targeted therapies to alter the count and function of T-cells in OMS are still poorly known. Mycophenolate mofetil, which exerts a cytostatic effect on T-cells, is used although not commonly [17]. Other cytostatic agents used in OMS include cyclophosphamide, methotrexate and mercaptopurine. Alkylating cyclophosphamide metabolites cause DNA fragmentation in nucleated cells, whereas methotrexate and mercaptopurine, which are antimetabolites, inhibit DNA synthesis leading to cell death. Therefore, both classes of drugs, exert their inhibitory effect on both T- and B-cells [1,9,10,15]. According to Pranzatelli et al. [15], due to the difficulty of identifying the mechanism which triggers OMS especially in recurrent cases, a combined treatment based on GS, ACTH, rituximab or IVIG and cyclophosphamide should be used.

Our experience with achieving a complete (4 cases) or partial (3 cases) response to the combined treatment with GS and cyclophosphamide without any noticeable adverse effects contributes to the existing body of evidence supporting the benefits of such treatment modality in OMS. Such children should be closely monitored since, as pointed out by Tayoshima et al. [2], despite a complete resolution of OMS, even a simple upper respiratory tract infection may trigger its recurrence. We have experienced recurrent opsoclonus twice in the same female after a burn and an infection.

Although a spontaneous remission of OMS can occur, an early combined treatment seems to be the most effective alternative, as it reduces cognitive impairment and morbidity in OMS [1,2,21]. As stated by Brunklaus et al. [22], over half of the children with OMS experiences suffer from a permanent intellectual and motor impairment. In our small sample, one child needs some additional support at school and two others stay hyperactive.

## 5. Conclusions

Neuroblastic tumour resection does not resolve OMS in children with OMS secondary to NT. The combined treatment with dexamethasone plus cyclophosphamide seems to be an effective and available treatment of OMS. Because of the small size of the group of children analysed, future observations and research are necessary.

## Supplementary Materials

The following are available online at https://www.mdpi.com/1010-660X/56/8/412/s1. Table S1: Characteristics of the examined group.
