# Supplementary material for: The Treatment of Opsoclonus-Myoclonus Syndrome Secondary to Neuroblastic Tumours—Single-Centre Experience and Literature Review
Source: Medicina (Kaunas). 2020 Aug 14;56(8):412. doi: 10.3390/medicina56080412 (PMC7466285; doi:10.3390/medicina56080412)
Supplement: Supplementary file 1 [file medicina-56-00412-s001.pdf]

Gender/ age (mths)	Tumor localization	Clinical stage	Histopathology	Duration of clinical symptoms OMS	Department first hospitalization	First signs	Therapy	Recent oncological status	Recent OMS status	Follow up (mths)
F/24	Suprarenal gland left	I	NBL differentiating type n-myc (-)	3 months	Neurology	opsoclony, myoclony, impaired balance, regression psycho-motoric development	1.resecion<br>2. GS (2 times)<br>3. IVIG<br>4.XVI courses CY+dexa	CR	PR (hyperactivity, intellectual disability)	170
F/36	Suprarenal gland right	I	NBL differentiating type n-myc (-)	1 week	Neurology	impaired balance, nystagmus	1.GS<br>2. resection<br>3. IVIG<br>4.XII courses CY+dexa	CR	CR	155
M/18	Suprarenal gland left	I	NBL differentiating type n-myc (-)	1 month	Neurology	myoclony, weakness of the lower limbs, problems with walking	1.resecion<br>2. GS<br>3. IVIG<br>4.XII courses CY+dexa	CR	PR (periodically myoclony after viral infections)	138
F/36	Paravertebral area L part	I	GNBL intermixed	3 months	Neurology	weakness of the lower limbs, problems with walking, dysarthry	1. GS<br>2. resection<br>3. XII courses CY+dexa	CR	PR (hyperactivity)	98
M/14	Suprarenal gland right	I	NBL differentiating type n-myc (-)	2 months	Neurology	regression psycho-motoric development, impaired balance, behavioral difficulties	1.resecion<br>2. GS<br>3. IVIG<br>4.XII courses CY+dexa<br>5. additionally 3 dexa pulses	CR	CR	87
F/36	Paravertebral area L part	III	NBL differentiating type n-myc (-)	1 month	Neurology	tremors of the upper limbs, impaired balance, behavioral difficulties	1. IVIG<br>2. partial resection<br>3. chemotherapy NBL (VP, CBDCA, CY, ADR, VCR)	CRu	CR	60
F/30	Paravertebral area Th part	I	GNBL intermixed	8 months	Neurology	nystagmus, problems with walking	1.resecion<br>2. GS<br>3. IVIG<br>4. VPA<br>4.XII courses CY+dexa<br>5. additionally 4 dexa pulses	CR	CR (but after therapy 2 times reactivation, after burns and viral infection)	16
